# Survey of owner motivations and veterinary input of owners feeding diets containing raw animal products

**DOI:** 10.7717/peerj.3031

**Published:** 2017-03-02

**Authors:** Stewart K. Morgan, Susan Willis, Megan L. Shepherd

**Affiliations:** 1 Department of Large Animal Clinical Sciences/Virginia-Maryland College of Veterinary Medicine, Virginia Polytechnic Institute and State University (Virginia Tech), Blacksburg, VA, United States; 2 Center for Survey Research, Virginia Polytechnic Institute and State University (Virginia Tech), Blacksburg, VA, United States

**Keywords:** Survey, Pet, Owner, Motivation, Resources, Raw, Diet, Meat, Veterinarians, Trust

## Abstract

**Background:**

The practice of feeding of diets containing raw animal products (RAP) to pets (dogs and cats) is discouraged by veterinary organizations and governmental public health organizations. Nevertheless, the practice of feeding RAP to pets is increasing in popularity. Pet owner motivations for feeding RAP diets to pets have not been explored and the benefits of RAP diets remain largely anecdotal. We hypothesized that pet owners feeding RAP diets would not rely on veterinary advice in choosing their pet’s diet. We also hypothesized that these owners would have lower levels of trust in veterinary advice with respect to nutrition relative to pet owners not feeding RAP.

**Methods:**

An anonymous web-based survey was developed to identify pet owner motivations for feeding RAP diets, and to characterize the veterinarian-client relationships of individuals feeding RAP diets.

**Results:**

There were 2,337 respondents and 2,171 completed surveys. Of survey respondents, 804 reported feeding RAP at the time of the survey. While 20% of pet owners feeding RAP relied on online resources to determine what or how much RAP to feed, only 9% reported consulting with a veterinarian in making decisions about feeding RAP. Pet owners feeding RAP reported lower levels of trust in veterinary advice both ‘in general’ and ‘with respect to nutrition’ than pet owners not feeding RAP. Most pet owners reported that a discussion regarding their pet’s nutrition does not occur at every veterinary appointment.

**Discussion:**

Pet owners feeding a RAP diet have lower trust in veterinary advice than pet owners not feeding a RAP diet. Owners feeding RAP are more reliant on online resources than their own veterinarian in deciding what and how much RAP to feed. Pet owners perceive that nutrition is not discussed at most veterinary appointments. Therefore, there is room for improvement in the veterinarian-client communication with regards to nutrition.

## Introduction

Raw animal product (RAP) diets are those containing portions of animal tissue, not subjected to heat or cooking during their preparation. These diets can contain muscle meat, organs (kidneys, liver, lung, spleen, etc.) and/or bone from poultry, mammals, or fish. The American Animal Hospital Association (AAHA), American Veterinary Medical Association (AVMA), and Food and Drug Administration (FDA) discourage the feeding of RAP diets to pets (AVMA, 2012; FDA, 2016). Nevertheless, the profit and market share from commercial RAP diets has grown rapidly in recent years (GfK, 2016) with retail sales of raw freeze-dried and raw frozen pet foods increasing 64% and 32%, respectively, between 2014 and 2015 (Tarkan, 2015).

Numerous studies (Freeman et al., 2013; Freeman, Janecko & Weese, 2013; Freeman & Michel, 2001; Kohler, Stengel & Neiger, 2012; Lenz et al., 2009; Leonard et al., 2011; Stiver et al., 2003; Strohmeyer et al., 2006) have reported the risks of feeding RAP diets, in particular bacterial disease, parasitic disease, and nutrient imbalance. Pathogenic *Salmonella* spp., *Listeria monocytogenes, Campylobacter* spp., *Clostridium* spp., and *Escherichia coli* (*E. coli)* have been associated with feeding RAP diets. Of these, *Salmonella* infections are most frequently documented. Pet dogs and cats are capable of carrying and shedding *Salmonella* spp. in their feces without clinical signs of illness (Carter & Quinn, 2000; Cobb & Stavisky, 2013; Finley et al., 2007; Joffe & Schlesinger, 2002; Leonard et al., 2011; Morley et al., 2006; Shimi & Barin, 1977). When clinical disease (salmonellosis) is present, clinical signs may include abortion, anorexia, conjunctivitis, gastroenteritis, hematochezia, and death (Carter & Quinn, 2000; Cobb & Stavisky, 2013). Animals most likely to contract salmonellosis from RAPs include the very young, older animals, and immunocompromised animals (Carter & Quinn, 2000; Cobb & Stavisky, 2013). Similarly, humans most at risk of infection from pathogenic bacteria found in RAP include children, the elderly, immunocompromised individuals, and pregnant women (Kendall et al., 2003; Leonard et al., 2011; Tam, Erebara & Einarson, 2010). Bacterial pathogens may be spread during diet handling and through environmental contamination from animals fed a RAP diet (FDA, 2016; Finley et al., 2007; Freeman et al., 2013; Freeman, Janecko & Weese, 2013; Joffe & Schlesinger, 2002; Lenz et al., 2009; Leonard et al., 2011; Morley et al., 2006; Nemser et al., 2014; Strohmeyer et al., 2006). In addition to zoonotic bacteria, pets fed raw meat may also contract zoonotic parasitic diseases (including toxoplasmosis and echinococcosis) (Araujo et al., 1975; Elmore et al., 2010; Jokelainen et al., 2012; Moro & Schantz, 2009; Schwabe et al., 1972; Strohmeyer et al., 2006). Raw diets (both commercial and homemade) may also be nutritionally inappropriate for the species or life-stage to which they are fed (Dillitzer, Becker & Kienzle, 2011; Freeman & Michel, 2001; Kerr et al., 2013; Kohler, Stengel & Neiger, 2012; Taylor et al., 2009; Zeugswetter, Vogelsinger & Handl, 2013).

Prior studies have reported that pet owners and dog breeders consider veterinarians their primary source for information regarding their pet’s nutrition and health care (Connolly, Heinze & Freeman, 2014; Freeman, Janecko & Weese, 2013; Kienzle, Bergler & Mandernach, 1998; Laflamme et al., 2008; Michel et al., 2008). However, less than half of dog breeders actually ask their veterinarian for nutritional advice (Connolly, Heinze & Freeman, 2014). Furthermore, pet owners that feed noncommercial diets are less likely to trust their veterinarian to provide sound nutritional advice (Connolly, Heinze & Freeman, 2014; Michel et al., 2008). Thus, while veterinarians are considered trusted and knowledgeable authorities on pet nutrition and healthcare, there remains a disconnect between what clients feed pets and the resources clients actually use in making diet choices. The possibility that owners feeding RAP diets are not discussing their pet’s nutrition with their veterinarian has not been explored.

Proponents of RAP diets claim that these diets are healthier and more natural for dogs and cats than conventional commercial pet foods (Billinghurst, 1993; Freeman et al., 2013; Lenz et al., 2009). Proponents of RAP diets have also touted these diets as “biologically appropriate” and have stated that dogs and cats “evolved to eat” RAP diets (BARFI World^®^, 2013; Beaton, 2016). The benefits claimed for RAP diets range from preservation of natural enzymes to elimination of artificial preservatives from pet’s diets.

Prior studies have not attempted to identify pet owner motivations for feeding RAP diets, and pet owner rationales for feeding RAP diets have been largely anecdotal (Freeman et al., 2013). Therefore, one goal of the study was identifying pet owner motivations for feeding RAP diets. A second goal was determining the degree of veterinary input, and trust in veterinarians, that RAP feeding pet owners have, relative to those that do not.

We hypothesized that pet owners feeding RAP diets discuss nutrition with their veterinarian less frequently than pet owners not feeding RAP diets. We also hypothesized that pet owners feeding RAP diets are less trusting of their veterinarian as a knowledgeable nutrition resource than pet owners not feeding a RAP diet. A greater comprehension of the motivations and resources that lead pet owners to feed RAP diets may help veterinarians communicate with clients that have chosen or that are considering to feed these diets.

## Materials & Methods

The results from this study are a subset of the results of a larger study that was designed to characterize the pets, pet owners, motivations for feeding RAP diets, and veterinarian-client relationships of individuals feeding RAP diets. An anonymous web-based survey was developed in conjunction with the Virginia Tech Center for Survey Research and disseminated to pet owners. Pet owners were recruited by word of mouth, posts on message boards, websites, and email contact. The email contacts were made via panel through the sampling firm Survey Sampling International and all respondents were directed to the survey link at the Virginia Tech Center for Survey Research. Any pet (cat and/or dog) owners living in the United States, and ≥18 years of age were eligible to participate. The survey was open between February 1, 2016 and April 1, 2016.

The web survey was developed in Qualtrics and hosted at the Virginia Tech Center for Survey Research. Many of the survey items were developed at the respondent level, meaning data were collected regarding the pet owner (survey respondent). For example, survey respondents were asked about their beliefs regarding a variety of dietary components, pet treatments, pet vaccinations and nutrition references. However, because there were multiple pets reported within individual households, many of the survey items were programmed within loops. Within each loop, the same data were collected for individual pets within a household. For example, the survey requested information on age, diet, and activities for each individual pet (both cats and dogs) in each respondent’s home. Therefore, throughout this document, the total number of responses reported for individual items reflects the varying number of responses provided to individual survey items (due to the varying number of cats and dogs within each household). Likewise, the varying response counts for individual items also reflects the fact that respondents were allowed to leave survey items blank unless the item was used as a determinant for subsequent questions being asked. For example, respondents were not allowed in the survey program to leave the item “Have you ever fed this [cat/dog] raw animal products one or more times weekly?” blank, because subsequent survey items that appeared were determined based on the respondent’s answer to this question.

Where statistically significant differences are reported, *P* < 0.05. Chi-square tests were used in determining statistically significant differences.

The experimental protocol was reviewed and approved by the institutional review board of Virginia Polytechnic Institute and State University (IRB# 16951).

## Results

### Survey participants

The survey generated 2,337 total respondents (Table 1). Most survey respondents were women, and the majority of respondents lived in a rural area. The largest percentage of respondents were between the ages of 51–60 years of age. While most owners owned both dogs and cats, more dog owners than cat owners are represented in the survey.

**Table 1:** Demographics of survey respondents.

Variable	Number of respondents	%
Partially completed survey	166	7.1
Completed survey	2,171	92.9
Cat owners (2,732 cats)	1,283	54.9
Dog owners (3,339 dogs)	1,680	71.9
**Gender**		
Female	1,776	82.3
Male	382	17.7
**Age (years)**		
18–30	332	15.3
31–40	368	17.0
41–50	404	18.7
51–60	676	31.3
>61	383	17.7
**Highest level of education**		
Did not complete high school or equivalent	30	1.4
High school or equivalent	323	14.9
Some college	492	22.7
Associates degree	274	12.7
Bachelor’s degree	591	27.3
Graduate degree	453	20.9
**Community type**		
Urban	507	23.5
Suburban	491	22.7
Rural	1,164	53.8

### Raw animal product feeding familiarity and implementation

The most common resource for first learning about feeding RAP, selected by both RAP feeders and non-RAP feeders, was the Internet (Table 2). The majority of dog (68%) and cat (65%) owners were familiar with the practice of feeding RAP diets. However, only 46% of dog owners and 38% of cat owners had actually fed RAP. Forty-two percent of all survey respondents fed RAP previously. Of those survey respondents that had fed RAP previously, 89% were feeding a RAP diet at the time of the survey. Forty-six percent of respondents who had never fed RAP were familiar with the practice of feeding RAP.

**Table 2:** Responses to the question “Where did you first learn about feeding raw animal product diets for pets?”

Resource	% Selected among respondents who feed RAP (*n* = 804)	% Selected among respondents who do not feed RAP (*n* = 1,270)
Internet	30	33
Friend/family member	19	26
Book, magazine, or printed source written by veterinarian other than your own	11	8
Book	10	3
Breeder instructions or advice	9	5
My veterinarian	8	7
Email group	6	3
Magazine	4	7
Pet store employee	3	8

### Characteristics of pet owners who feed raw animal products

Ninety-three percent of respondents who feed their pets RAP were online at least one hour per day, with 60 percent online more than 2 h per day. The majority of RAP feeders in the survey (89%) were female. 39 percent of RAP feeders were 40 years of age or younger and 61% were 41 years of age or older. The majority of RAP feeders (61%) did not have children. More than half (54%) had at least a Bachelor’s degree. Almost half (48%) lived in a suburban area or small town/city, with 24% residing in the countryside (rural area) and 28% residing in an urban area.

### Decision making and raw animal product feeding

Differences were detected in the reasons for why pets are fed RAP (Table 3). The most common reason for cat owners to start feeding their cat RAP was “getting information from a veterinary resource (these resources included books, magazine articles, online resources, and discussions with veterinarians associated with companies producing raw diets) other than their veterinarian.” The next most common reason for cat owners electing to feed RAP was that they “try not to consume processed foods and do not want their cat to consume them either.” Among dog owners, the two most common reasons for electing to feed their dog RAP were that a RAP diet ‘is healthier’ and ‘more natural.’

**Table 3:** Reasons for electing to feed raw animal products. Percentages for which each of the reasons for feeding RAP was reported by dog and cat owners feeding a RAP diet. Respondents were allowed to select more than one reason for electing to feed their pets a RAP diet.

Cause for electing to feed pet rap	Rap fed cats (*n* = 765) owner responses (%)	Rap fed dogs (*n* = 1,598) owner responses (%)
Concern about safety/quality control/nutritional value of commercial foods	37.5	67.3
Discussion with a friend or family member	49.8	24.0
Read about raw animal products (RAP) on a message board or website	53.9	30.0
My veterinarian	58.2	13.8
A veterinary resource other than my veterinarian	78.0	22.0
I try not to consume processed foods and do not want my pet to consume them either	72.3	35.2
To prevent food allergies	44.1	41.5
To improve dental or oral hygiene	29.9	66.5
To improve the skin or coat	31.9	67.5
To improve my pet’s immune system	68.0	65.7
Feeding a raw animal product diet is healthier	15.4	77.4
Feeding a raw animal product diet is more natural	40.0	71.2
My pet prefers a raw animal product diet	18.0	49.9
Other reason	25.9	23.8

The most prevalent information source utilized by RAP feeders for deciding what or how much RAP to feed were online resources (20% of RAP feeders). The second most prevalent source of information were published references (14% of RAP feeders). Less than 10% of RAP feeders (9%) reported consulting with a nutritionist or veterinarian in making decisions about feeding RAP.

### Beliefs regarding pet health

Pet owners feeding RAP were less likely to find vaccinations and parasite screening and prevention to be of benefit to their pet than owners not feeding RAP (Table 4).

**Table 4:** Respondents considering vaccination or parasite prevention and screening beneficial.

Pet health variable	All respondents %	Do not feed rap (%)	Feed rap (%)
Vaccination^*^	74	78	51
Flea & tick prevention^*^	75	83	60
Deworming & heartworm prevention^*^	83	92	72
Routine worm & other parasite screening	86	90	82

**Notes.**

* Significant difference between RAP feeders and non-RAP feeders.

Overall, dog owners were more likely to take their dogs to a veterinarian more than once a year than cat owners (Tables 5 and 6). Cat owners who feed RAP diets were slightly more likely to take their cat to a veterinarian than non-RAP feeders. On the other hand, dog owners who feed RAP diets were slightly less likely to take their dog to a veterinarian than non-RAP feeders.

**Table 5:** Frequency of veterinary visits for cats fed raw animal products and cats not fed raw animal products.

How often do you take this cat to a veterinarian?	Fed rap (%)	Not fed rap (%)	All cats (%)
Never, no veterinarian	6.1	11.6	10.0
Less than once a year	23.1	25.9	24.8
About once a year	47.1	48.9	48.0
More than once a year	23.7	13.6	17.2
Total cats	903	1,709	2,644

**Table 6:** Frequency of veterinary visits for dogs fed raw animal products and dogs not fed raw animal products.

How often do you take this dog to a veterinarian?	Fed rap (%)	Not fed rap (%)	All dogs (%)
Never, no veterinarian	3.6	5.4	4.5
Less than once a year	18.2	11.4	15.2
About once a year	46.1	52.2	48.9
More than once a year	32.1	31.0	31.4
Total dogs	1,761	1,466	3,254

### Nutrition discussions with pet’s veterinarian among pet owners feeding raw animal products and pet owners not feeding raw animal products

A discussion regarding nutrition at every veterinary visit was reported for only 35% (*n* = 1,908 of 5,429) of all pets in the survey and nutrition was never discussed at veterinary visits for 20% (*n* = 1,072) of pets in the survey. RAP feeders were less likely than non-RAP feeders to say they “never” discuss their pet’s diet/nutrition with their veterinarian. However, there are differences with regard to the frequency of such discussions among cat and dog owners. Specifically, more cat owners feeding RAP reported discussing nutrition with their veterinarian each visit than cat owners who do not feed RAP (43% vs. 35%), while slightly fewer dog owners feeding a RAP diet reported discussing nutrition with their veterinarian each visit than dog owners not feeding a RAP diet (32% vs. 35%). Fewer cat owners feeding RAP reported only discussing nutrition at veterinary visits when they initiate the discussion with their veterinarian than cat owners that do not feed RAP (10% vs. 15%). Similarly, fewer dog owners who feed RAP reported only discussing nutrition at veterinary visits when they initiate the discussion with their veterinarian than dog owners that do not feed RAP (15% vs. 21%). Both dog and cat owners that feed RAP diets were more likely than non-RAP feeders to say that they only discuss pet nutrition with their veterinarian (or veterinary health team member) when asked by their veterinarian (or veterinary health team member).

### Findings related to trust of veterinarians

There were significant differences in the levels of trust regarding the advice of veterinarians among RAP feeders and pet owners who do not feed RAP. Specifically, non-RAP feeders are more likely to say they trust the advice of veterinarians, in general, “very much” than RAP feeders (Table 7).

**Table 7:** General trust in veterinary advice among pet owners feeding raw animal products and pet owners that do not feed raw animal products.

How much do you trust the advice of veterinarians in general?	Feed rap (%)	Do not feed rap (%)
Very much	36.2	63.9
Somewhat	50.7	31.3
A little	8.3	4.0
Not very much	4.8	0.7
Total respondents	895	1,251

The findings regarding trust in veterinary advice, in general, were highly similar among cat and dog owners (Table 8).

**Table 8:** General trust in veterinary advice among cat owners and dog owners.

How much do you trust the advice of veterinarians in general?	Cat owners (%)	Dog owners (%)
Very much	53.1	51.7
Somewhat	39.0	39.6
A little	5.6	6.2
Not very much	2.3	2.5
Total respondents	1,204	1,604

RAP feeders trusted veterinarians less, as a knowledgeable resource regarding pet nutrition, than non-RAP feeders (Table 9). However, trust levels among cat and dog owners were similar, with regard to veterinarians as a knowledgeable resource regarding pet nutrition (Table 10).

**Table 9:** Trust in veterinarians as a knowledgeable resource with respect to pet nutrition among pet owners that feed raw animal products and pet owners that do not feed raw animal products.

With respect to pet nutrition, to what extent do you trust veterinarians as a knowledgeable resource?	Feed rap (%)	Do not feed rap (%)
Very much	12.7	54.5
Somewhat	32.4	37.0
A little	15.8	5.6
Not very much	39.2	2.9
Total respondents	893	1,249

**Table 10:** Trust in veterinarians as a knowledgeable resource with respect to pet nutrition among cat owners and dog owners.

With respect to pet nutrition, to what extent do you trust veterinarians as a knowledgeable resource?	Cat owners (%)	Dog owners (%)
Very much	38.6	35.9
Somewhat	34.1	34.9
A little	9.5	10.3
Not very much	17.9	18.9
Total respondents	1,203	1,601

## Discussion

Survey respondents feeding RAP diets trust veterinarians less and rely heavily on online resources in determining what to feed their pets. RAP feeders had lower confidence in veterinarians and veterinary advice than non-RAP feeders. A majority of pet owners reported that they trust veterinarians as a knowledgeable resource with respect to pet nutrition either “very much” or “somewhat.” Most pet owners feeding RAP reported trusting veterinarians either “not very much” or “a little” with respect to pet nutrition. Similarly, previous studies have reported that pet owners that do not feed commercial diets have less trust in veterinarians, as a source of information on pet nutrition, than those that feed commercial diets (Connolly, Heinze & Freeman, 2014; Rajagopaul et al., 2016). Prior studies have reported that clients consider veterinarians to be their principle or most trustworthy source of information regarding pet nutrition (Connolly, Heinze & Freeman, 2014; Freeman, Janecko & Weese, 2013; Kienzle, Bergler & Mandernach, 1998; Laflamme et al., 2008; Rajagopaul et al., 2016), however only 9% of RAP feeders in this study reported that a veterinarian or animal nutritionist was their primary resource regarding their pet’s nutrition. It has been reported that approximately 15–17% of pet owners use online resources as a primary source of information for obtaining information on pet nutrition (Freeman, Janecko & Weese, 2013; Laflamme et al., 2008). Thus, the overall percentage of RAP feeders that used online resources as their primary source of information for pet nutrition (20%) was similar between this study and prior studies.

Cat owners feeding RAP diets had lower levels of trust for veterinarians as a source of information for pet nutrition. However, the top reason these owners elected to feed a RAP diet was by obtaining information from a veterinary resource other than their veterinarian. Resources used included feline nutrition websites (authored by veterinarians) and books on raw feeding (authored by veterinarians). Interestingly, the majority (58%) of cat owners feeding RAP diets reported that they decided to feed a RAP diet on their own veterinarian’s advice. Paradoxically, cat owners feeding RAP diets were more likely to take their cat to a veterinarian than those not feeding RAP diets, and were more likely to discuss their pet’s nutrition with their veterinarian at each visit. These data indicate that the population of cat owners that are feeding RAP diets is different from the population of dog owners that feed RAP diets. For example, concern about commercial pet food quality and safety was not a primary factor in cat owners’ decision to feed a RAP diet. For cat owners, discussions with friends and family members, their own veterinarian, veterinary resources, and their personal beliefs in not eating processed foods played key roles in their desire to feed a RAP diet.

These data suggest dog owners feeding RAP diets appear to make the decision to feed a RAP diet with pet health as a primary motivating factor. This is paradoxical, as this group of pet owners had a greater degree of mistrust of veterinarians and veterinary advice with respect to nutrition than those not feeding RAP diets. Thus, dog owners feeding RAP diets appear to trust online information and information from non-veterinary animal health and nutrition resources more than they trust veterinary resources. A prior study (Lenz et al., 2009) also indicated that there were significant differences between dog owners that feed raw meat to their pets and those that do not. In that study, unlike owners that did not feed raw meat, owners feeding raw meat tended to believe that raw meat was healthy for their pet, and chose their pet’s diet based on the perceptions that diet can help prevent diseases, a food’s perceived nutritional value, and the desire to avoid preservatives. Dogs belonging to owners feeding raw meat in this study were significantly more likely to have not been vaccinated in the prior three years (Lenz et al., 2009).

Although the reasons for the differences between the factors motivating cat and dog owners to feed RAP diets are unclear, the nature of the relationships between these owners and their pets may play a role. Prior studies have found differences between pet owners that feed an inappropriate diet and the relationships they have with their pets compared to those feeding an appropriate diet. For example, owners of overweight cats and obese dogs have both been reported to “over-humanize” their pets. On the other hand, while owners of overweight cats were reported to have a close relationship with their pets, this was not the case with the owners of obese dogs (Kienzle & Bergler, 2006; Kienzle, Bergler & Mandernach, 1998). Pet owner perceptions of their own personal health may also play an indirect role in pet diet. For example, owners of overweight cats had a greater concern for their own health than owners of cats with normal body weight (Kienzle & Bergler, 2006), while this was not the case with owners of obese dogs (Kienzle, Bergler & Mandernach, 1998). The reasons for the differences between the factors motivating cat and dog owners to feed RAP diets will be further explored.

These data reveal a low level of veterinary communication with clients with respect to nutrition. Nevertheless, the majority of respondents reported trusting veterinarians either “very much” or “somewhat” with respect to pet nutrition. These data are similar to previous studies, which have reported that most veterinarians do not discuss nutrition with clients at each veterinary visit or that, while veterinarians are considered trusted resources, with respect to pet nutrition, they are often not consulted (Connolly, Heinze & Freeman, 2014; Rajagopaul et al., 2016).

For RAP diet feeders, the low level of trust in veterinary advice, in general, and the high level of distrust of veterinarians as a resource for pet nutrition, may be an indication of poor communication between veterinarians and these clients. Veterinary disapproval (implicit or explicit), and the veterinarian’s method of communicating that disapproval, of a client’s decision to feed RAP diets (or other alternative diets) to their pet may negatively impact client trust in their veterinarian, with respect to their pet’s nutrition. Clients have been reported to have greater compliance with veterinary recommendations when there is a strong veterinarian-client bond (Lue, Pantenburg & Crawford, 2008). The most crucial part a strong veterinarian-client bond is communication (Lue, Pantenburg & Crawford, 2008). A survey published in 2013 reported that, for equine veterinarians, the most common reasons for not routinely providing nutritional advice to clients were client noncompliance/disinterest (56%), the belief that they lacked sufficient knowledge to provide nutritional advice to clients (38%), and insufficient time (6%) (Roberts & Murray, 2013).

While client noncompliance has been found to be related to the strength of communication and the veterinarian-client relationship, a key factor in client perceptions of the quality of communication is the ability of veterinarians to provide thorough recommendations and options (Lue, Pantenburg & Crawford, 2008). The manner in which veterinarians pose questions in obtaining a diet history from clients has also been found to impact the completeness and accuracy of the information obtained (MacMartin et al., 2015).

The ability to adequately both provide nutrition recommendations and to explain the rationale for those recommendations is likely linked to a veterinarian’s nutritional competency. In the aforementioned 2013 study, most of these survey respondents reported low levels of confidence in equine nutrition upon graduation from their veterinary college (Roberts & Murray, 2013; Roberts & Murray, 2014). Similar perceptions of low levels of confidence in both large and small animal veterinary nutrition training by veterinary colleges have been reported in surveys of recent graduates in the United States (Buffington & LaFlamme, 1996; Roberts & Murray, 2014). A survey of deans and faculty members from 63 European veterinary colleges revealed that while 97% of respondents agreed that a core competency of recent graduates was the ability to perform a nutritional assessment on patients, only 41% of the respondents reported that they were satisfied with the skills and performance of their school’s graduates in veterinary nutrition (Becvarova et al., 2016). Paradoxically, approximately half of the schools surveyed did not have nutrition rounds or rotations for final year students and were not planning on doing so.

Currently, only 50% of veterinary colleges within in the United States have a boarded veterinary nutritionist (Becvarova et al., 2016). All schools receiving accreditation (or provisional accreditation) in the United States since 2010 lack boarded (ACVN or ECVN) veterinary nutritionists. The majority of AVMA Council of Education accredited veterinary colleges outside the United States lack boarded (ACVN or ECVN) veterinary nutritionists. While non-profit organizations (such as the Mark Morris Institute) provide educational resources to many veterinary colleges, many veterinary colleges do not use these resources or do not provide any training in nutrition for final year veterinary students. Pet food companies are also an important resource for veterinary students and veterinary practitioners. Not only do these companies perform pet nutrition research, they also provide educational resources to veterinary students and practitioners (including supporting continuing education).

The results from this survey reveal that there is a need for veterinarians to discuss nutrition with clients and that veterinarians must be able to competently explain to clients their reasons for making nutrition recommendations. Veterinarians were not the primary source from which clients were first introduced to the practice of feeding RAP diets, however veterinarians are generally client’s most trusted resource with respect to pet health and nutrition. These data indicate that clients that choose to feed raw are relying more on online resources which may or may not provide accurate information to pet owners. Veterinarians have a duty to protect animal health and welfare and to promoting public health. This entails being capable of effectively communicating information regarding animal nutrition and husbandry with clients.

As in prior surveys of pet owners in which respondent gender was reported (Connolly, Heinze & Freeman, 2014; Freeman, Janecko & Weese, 2013; Heuberger & Wakshlag, 2011; Hutchinson et al., 2011; Kienzle & Bergler, 2006; Rajagopaul et al., 2016), this survey’s respondents were primarily female. The reason for this bias is unclear, considering that these survey’s populations were different and the surveys had different goals. Nevertheless, the results from this survey do reveal differences in both owner motivations and veterinary input between RAP feeding pet owners.

## Conclusions

Dog and cat owners have different motivations for feeding a RAP diet, with dog owners reporting pet health as a primary motivation and cat owners reporting use of a veterinary resource other than their own veterinarian or their personal aversion to processed foods as primary motivations for feeding RAP. An association between RAP feeding and reduced trust of veterinary advice (in particular, nutritional advice) was noted, indicating probable deficits in the veterinarian-client relationship in RAP feeding clients. Furthermore, most respondents reported that their veterinarian did not discuss their pet’s nutrition with them at each visit. These results may indicate deficiencies in veterinarian’s comfort with discussing nutrition with their clients, which may be a consequence of insufficient/inadequate training in nutrition during veterinary school and from continuing education following graduation. Clients feeding a RAP diet are clients whose pets may benefit from attempts by their veterinarian to improve communication and nutritional competency.

##  Supplemental Information

10.7717/peerj.3031/supp-1 Supplemental Information 1 Raw data

## Additional Information and Declarations

### Data Availability

The following information was supplied regarding data availability: The raw data has been supplied as Data S1.

## References

[ref-1] Araujo FP, Schwabe CW, Sawyer JC, Davis WG (1975). Hydatid disease transmission in California. A study of the Basque connection. American Journal of Epidemiology.

[ref-2] AVMA (2012). Raw or undercooked animal-source protein in cat and dog diets. https://www.avma.org/KB/Policies/Pages/Raw-or-Undercooked-Animal-Source-Protein-in-Cat-and-Dog-Diets.aspx.

[ref-3] BARFI World^®^ (2013). What is BARF?^®^. http://www.barfworld.com/html/learn_more/what_is_barf.shtml.

[ref-4] Beaton L (2016). Champion Petfoods: specialists in biologically appropriate dog, cat food. http://www.petfoodindustry.com/articles/4485-raw-petfood-a-growing-market-segment-in-spite-of-controversy.

[ref-5] Becvarova I, Prochazka D, Chandler ML, Meyer H (2016). Nutrition education in European veterinary schools: are European veterinary graduates competent in nutrition?. Journal of Veterinary Medical Education.

[ref-6] Billinghurst I (1993). Give your dog a bone: the practical commonsense way to feed dogs for a long healthy life.

[ref-7] Buffington CA, LaFlamme DP (1996). A survey of veterinarians’ knowledge and attitudes about nutrition. Journal of the American Veterinary Medical Association.

[ref-8] Carter ME, Quinn PJ, Wray C, Wray A (2000). *Salmonella* infections in dogs and cats. Salmonella in domestic animals.

[ref-9] Cobb MA, Stavisky J, Barrow PA, Methner U (2013). *Salmonella* in dogs and cats. Salmonella in domestic animals.

[ref-10] Connolly KM, Heinze CR, Freeman LM (2014). Feeding practices of dog breeders in the United States and Canada. Journal of the American Veterinary Medical Association.

[ref-11] Dillitzer N, Becker N, Kienzle E (2011). Intake of minerals, trace elements and vitamins in bone and raw food rations in adult dogs. British Journal of Nutrition.

[ref-12] Elmore SA, Jones JL, Conrad PA, Patton S, Lindsay DS, Dubey JP (2010). Toxoplasma gondii: epidemiology, feline clinical aspects, and prevention. Trends in Parasitology.

[ref-13] FDA (2016). Get the facts! Raw pet food diets can be dangerous to you and your pet. http://www.fda.gov/AnimalVeterinary/ResourcesforYou/AnimalHealthLiteracy/ucm373757.htm.

[ref-14] Finley R, Ribble C, Aramini J, Vandermeer M, Popa M, Litman M, Reid-Smith R (2007). The risk of *salmonellae* shedding by dogs fed *Salmonella*-contaminated commercial raw food diets. The Canadian Veterinary Journal.

[ref-15] Freeman LM, Chandler ML, Hamper BA, Weeth LP (2013). Current knowledge about the risks and benefits of raw meat-based diets for dogs and cats. Journal of the American Veterinary Medical Association.

[ref-16] Freeman LM, Janecko N, Weese JS (2013). Nutritional and microbial analysis of bully sticks and survey of opinions about pet treats. The Canadian Veterinary Journal.

[ref-17] Freeman LM, Michel KE (2001). Evaluation of raw food diets for dogs. Journal of the American Veterinary Medical Association.

[ref-18] GfK (2016). Pet food category insights. http://www.gfk.com/landing-pages/landing-pages-us/pet-insight-scoop/pet-food-category-insights/.

[ref-19] Heuberger R, Wakshlag J (2011). The relationship of feeding patterns and obesity in dogs. Journal of Animal Physiology and Animal Nutrition.

[ref-20] Hutchinson D, Freeman LM, Schreiner KE, Terkla GD (2011). Survey of opinions about nutritional requirements of senior dogs and analysis of nutrient profiles of commercially available diets for senior dogs. International Journal of Applied Research in Veterinary Medicine.

[ref-21] Joffe DJ, Schlesinger DP (2002). Preliminary assessment of the risk of *Salmonella* infection in dogs fed raw chicken diets. The Canadian Veterinary Journal.

[ref-22] Jokelainen P, Simola O, Rantanen E, Nareaho A, Lohi H, Sukura A (2012). Feline toxoplasmosis in Finland: cross-sectional epidemiological study and case series study. Journal of Veterinary Diagnostic Investigation.

[ref-23] Kendall P, Medeiros LC, Hillers V, Chen G, DiMascola S (2003). Food handling behaviors of special importance for pregnant women, infants and young children, the elderly, and immune-compromised people. Journal of the American Dietetic Association.

[ref-24] Kerr KR, Beloshapka AN, Morris CL, Parsons CM, Burke SL, Utterback PL, Swanson KS (2013). Evaluation of four raw meat diets using domestic cats, captive exotic felids, and cecectomized roosters. Journal of Animal Science.

[ref-25] Kienzle E, Bergler R (2006). Human-animal relationship of owners of normal and overweight cats. The Journal of Nutrition.

[ref-26] Kienzle E, Bergler R, Mandernach A (1998). A comparison of the feeding behavior and the human–animal relationship in owners of normal and obese dogs. The Journal of Nutrition.

[ref-27] Kohler B, Stengel C, Neiger R (2012). Dietary hyperthyroidism in dogs. Journal of Small Animal Practice.

[ref-28] Laflamme DP, Abood SK, Fascetti AJ, Fleeman LM, Freeman LM, Michel KE, Bauer C, Kemp BL, Doren JR, Willoughby KN (2008). Pet feeding practices of dog and cat owners in the United States and Australia. Journal of the American Veterinary Medical Association.

[ref-29] Lenz J, Joffe D, Kauffman M, Zhang Y, LeJeune J (2009). Perceptions, practices, and consequences associated with foodborne pathogens and the feeding of raw meat to dogs. The Canadian Veterinary Journal.

[ref-30] Leonard EK, Pearl DL, Finley RL, Janecko N, Peregrine AS, Reid-Smith RJ, Weese JS (2011). Evaluation of pet-related management factors and the risk of *Salmonella* spp. carriage in pet dogs from volunteer households in Ontario (2005–2006). Zoonoses and Public Health.

[ref-31] Lue TW, Pantenburg DP, Crawford PM (2008). Impact of the owner-pet and client-veterinarian bond on the care that pets receive. Journal of the American Veterinary Medical Association.

[ref-32] MacMartin C, Wheat HC, Coe JB, Adams CL (2015). Effect of question design on dietary information solicited during veterinarian-client interactions in companion animal practice in Ontario, Canada. Journal of the American Veterinary Medical Association.

[ref-33] Michel KE, Willoughby KN, Abood SK, Fascetti AJ, Fleeman LM, Freeman LM, Laflamme DP, Bauer C, Kemp BL, Doren JR (2008). Attitudes of pet owners toward pet foods and feeding management of cats and dogs. Journal of the American Veterinary Medical Association.

[ref-34] Morley PS, Strohmeyer RA, Tankson JD, Hyatt DR, Dargatz DA, Fedorka-Cray PJ (2006). Evaluation of the association between feeding raw meat and *Salmonella* enterica infections at a Greyhound breeding facility. Journal of the American Veterinary Medical Association.

[ref-35] Moro P, Schantz PM (2009). Echinococcosis: a review. International Journal of Infectious Diseases.

[ref-36] Nemser SM, Doran T, Grabenstein M, McConnell T, McGrath T, Pamboukian R, Smith AC, Achen M, Danzeisen G, Kim S, Liu Y, Robeson S, Rosario G, McWilliams Wilson K, Reimschuessel R (2014). Investigation of Listeria, *Salmonella*, and toxigenic *Escherichia coli* in various pet foods. Foodborne Pathogens and Disease.

[ref-37] Rajagopaul S, Parr JM, Woods JP, Pearl DL, Coe JB, Verbrugghe A (2016). Owners’ attitudes and practices regarding nutrition of dogs diagnosed with cancer presenting at a referral oncology service in Ontario, Canada. Journal of Small Animal Practice.

[ref-38] Roberts JL, Murray JA (2013). Survey of equine nutrition: perceptions and practices of veterinarians in Georgia, USA. Journal of Equine Veterinary Science.

[ref-39] Roberts JL, Murray JA (2014). Equine nutrition in the United States: a review of perceptions and practices of horse owners and veterinarians. Journal of Equine Veterinary Science.

[ref-40] Schwabe CW, Ruppanner R, Miller CW, Fontaine RE, Kagan IG (1972). Hydatid disease is endemic in California. California Medicine.

[ref-41] Shimi A, Barin A (1977). *Salmonella* in cats. Journal of Comparative Pathology.

[ref-42] Stiver SL, Frazier KS, Mauel MJ, Styer EL (2003). Septicemic salmonellosis in two cats fed a raw-meat diet. Journal of the American Animal Hospital Association.

[ref-43] Strohmeyer RA, Morley PS, Hyatt DR, Dargatz DA, Scorza AV, Lappin MR (2006). Evaluation of bacterial and protozoal contamination of commercially available raw meat diets for dogs. Journal of the American Veterinary Medical Association.

[ref-44] Tam C, Erebara A, Einarson A (2010). Food-borne illnesses during pregnancy: prevention and treatment. Canadian Family Physician.

[ref-45] Tarkan L (2015). Raw pet food sales are booming, but are the products safe?. http://fortune.com/2015/08/06/raw-pet-food-sales-safety/.

[ref-46] Taylor MB, Geiger DA, Saker KE, Larson MM (2009). Diffuse osteopenia and myelopathy in a puppy fed a diet composed of an organic premix and raw ground beef. Journal of the American Veterinary Medical Association.

[ref-47] Zeugswetter FK, Vogelsinger K, Handl S (2013). Hyperthyroidism in dogs caused by consumption of thyroid-containing head meat. Schweizer Archiv für Tierheilkunde.

